# *Trans*-Cinnamaldehyde Eluting Porous Silicon Microparticles Mitigate Cariogenic Biofilms

**DOI:** 10.3390/pharmaceutics14071428

**Published:** 2022-07-07

**Authors:** Afreen Jailani, Shanthini Kalimuthu, Vidhyashree Rajasekar, Sumanta Ghosh, Pierre-Yves Collart-Dutilleul, Naveen Fatima, Hyun Koo, Adline Princy Solomon, Frederic Cuisinier, Prasanna Neelakantan

**Affiliations:** 1 Division of Restorative Dental Sciences, Faculty of Dentistry, The University of Hong Kong, Hong Kong SAR, China; afreenjailani@gmail.com (A.J.); shan97@connect.hku.hk (S.K.); vidhya@connect.hku.hk (V.R.); u3008499@connect.hku.hk (S.G.); 2 Quorum Sensing Laboratory, Centre for Research in Infectious Diseases, School of Chemical and Biotechnology, SASTRA Deemed University, Thanjavur 613401, India; adlineprinzy@sastra.ac.in; 3 Laboratory of Bioengineering and Nanosciences, University of Montpellier, CEDEX 5, 34193 Montpellier, France; pierre-yves.collart-dutilleul@umontpellier.fr (P.-Y.C.-D.); naveen.fatima@etu.umontpellier.fr (N.F.); frederic.cuisinier@umontpellier.fr (F.C.); 4 Biofilm Research Labs, Department of Orthodontics, Divisions of Pediatric Dentistry and Community Oral Health, School of Dental Medicine, University of Pennsylvania, Philadelphia, PA 19104, USA; koohy@upenn.edu

**Keywords:** acidogenicity, biofilm, caries, trans-cinnamaldehyde

## Abstract

Dental caries, a preventable disease, is caused by highly-adherent, acid-producing biofilms composed of bacteria and yeasts. Current caries-preventive approaches are ineffective in controlling biofilm development. Recent studies demonstrate definite advantages in using natural compounds such as trans-cinnamaldehyde in thwarting biofilm assembly, and yet, the remarkable difficulty in delivering such hydrophobic bioactive molecules prevents further development. To address this critical challenge, we have developed an innovative platform composed of components with a proven track record of safety. We fabricated and thoroughly characterised porous silicon (pSi) microparticles to carry and deliver the natural phenyl propanoid trans-cinnamaldehyde (TC). We investigated its effects on preventing the development of cross-kingdom biofilms (*Streptococcus mutans* and *Candida albicans*), typical of dental caries found in children. The prepared pSi microparticles were roughly cubic in structure with 70–75% porosity, to which the TC (pSi-TC) was loaded with about 45% efficiency. The pSi-TC particles exhibited a controlled release of the cargo over a 14-day period. Notably, pSi-TC significantly inhibited biofilms, specifically downregulating the glucan synthesis pathways, leading to reduced adhesion to the substrate. Acid production, a vital virulent trait for caries development, was also hindered by pSi-TC. This pioneering study highlights the potential to develop the novel pSi-TC as a dental caries-preventive material.

## 1. Introduction

The oral cavity is home to several bacteria and fungi, which include both beneficial and harmful species. However, the formation of a highly adherent plaque biofilm with an increase in pathogenic species (dysbiosis) results in costly diseases such as dental caries [1,2]. Fluoride compounds are the most-used caries-preventive agents due to their effects on the remineralisation of dental hard tissues. Unfortunately, fluorides only have limited effectiveness against biofilms [3]. More importantly, fluoride-resistant microbial strains have already emerged, adding to the global challenge of antimicrobial resistance [4]. On the other hand, antiseptics such as chlorhexidine potentially amplify dysbiosis by their broad-spectrum microbicidal effects [5,6]. Therefore, there is a critical need to develop novel, non-microbicidal strategies that can inhibit biofilm development and caries-related virulence [7].

Trans-Cinnamaldehyde (TC) is a bioactive flavonoid with antimicrobial activity against several bacterial and fungal species [8,9,10,11,12,13]. We recently demonstrated that TC reduced biofilm development in Gram-positive bacteria such as *Streptococcus mutans* [11] and *Enterococcus faecalis* [12] monocultures. TC can destroy the polysaccharide component of the cell wall thereby affecting the cell membrane integrity and cell division. However, we and others have reported that a sub-inhibitory concentration of TC inhibits quorum sensing in bacteria and thereby inhibits several quorum sensing-mediated virulence phenomena (including adhesion, biofilm formation, and the reduced production of extracellular polymeric matrices) in Gram-positive bacteria such as *Streptococcus mutans* and *Enterococcus faecalis* [8,9,10,11,12,13]. However, the major challenge in using TC is its hydrophobicity, which impedes its availability in biofilms, requiring rather high concentrations (500 µg/mL) for the biofilm-inhibitory effects [11]. Therefore, it is critical to develop novel delivery methods that can enhance the biofilm-inhibitory effects of TC.

Micro and nanoparticles are efficient methods of increasing the availability of water-insoluble or sparingly soluble bioactive molecules. Such particles can be fine-tuned to allow for the controlled release of the drug and elicit target-specific action, while dramatically reducing the concentration of the drugs required for their action [14]. Porous silicon (pSi) particles are a combination of oxygen (53.3%) and silicon (46.8%) [15,16]. They are considered excellent drug carriers due to their favourable properties including large surface area, easily modifiable surface, and tunable pore size [17]. Additionally, the remarkable biocompatibility of pSi and its tunable resorbability mean it is an ideal candidate for drug delivery, making it useful in a variety of medical applications [18]. Despite such promising properties, pSi remains to be exploited as a carrier of natural antibiofilm molecules. Once developed, this drug delivery platform can be readily incorporated into varnishes and pit and fissure sealants in high-risk areas to prevent cariogenic biofilm development.

*Streptococcus mutans* is the primary driver of the caries process as it produces acids which cause demineralization of the dental hard tissues [19]. Being acidogenic and aciduric, it shifts the homeostasis towards a cariogenic environment at the tooth-biofilm interface [20]. Such a problem is also common at the interface between the tooth and restorations, resulting in secondary caries. Emerging evidence strongly suggests that cross-kingdom synergy between *S. mutans* and the yeast *Candida albicans* results in an increase in the cariogenic virulence of plaque [21,22,23] and this cross-kingdom biofilm is typical of dental caries in toddlers and pre-school children [24]. While some studies have addressed the inhibition of such cross-kingdom biofilms, no natural compound-based strategy with an enhanced delivery approach has been developed to inhibit the development and virulence of this cross-kingdom biofilm.

Therefore, the aim of this study was to encapsulate TC in pSi particles and examine its efficiency in preventing the development of cross-kingdom biofilms (*S. mutans* + *C. albicans*). We tested the hypothesis that TC-eluting pSi particles (pSi-TC) can prevent the development of biofilms and inhibit its caries-related virulence phenotype (acid production).

## 2. Materials and Methods

### 2.1. Synthesis and Characterization of pSi Particles

Porous silicon wafers were prepared by the electrochemical etching of crystalline silicon wafers (p++ type boron-doped crystalline, 0.0012 Ω-cm resistivity) in a custom-made Teflon cell. The etching was performed under a current density of 200 mA/cm^2^ for 30 min in hydrofluoric acid solution in ethanol (3:1 HF: ethanol, *v*/*v*) to create a porous structure. This porous layer was detached from the bulk wafer with a second etching in 3.1% HF in ethanol, at a current intensity of 4 mA/cm^2^ for 4 min. The detached layer was rinsed with ethanol and subjected to sonication for 5 min at 25 Hz in an ultrasonic bath. All particles were thermally oxidized for 1 h at 400 °C to slow the particle resorption in aqueous solution.

**Figure 1:**
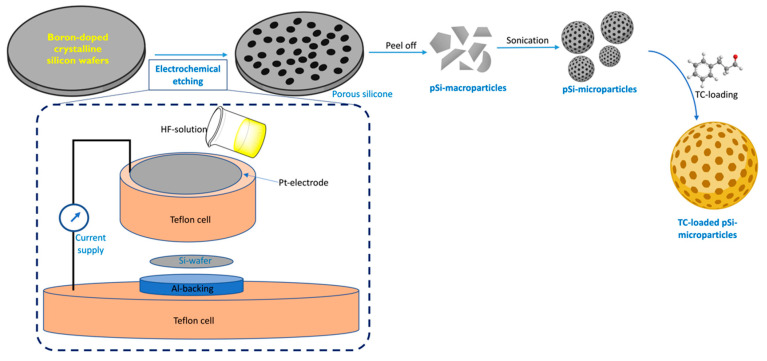
Schematic illustrating the synthesis of the pSi-TC microparticles.

The microparticles were sterilized with 70% ethanol for 10 min before drying under sterile airflow. The pSi particles were analyzed by scanning electron microscopy (SEM) (Analytic FEI Quanta FEG 200) to determine the particle size and pore diameter. An acceleration voltage of 20.00 kV was used at a pressure of 0.5 Torr. Global porosity was determined by interferometric reflectance spectroscopy (IRS), using white light at normal incidence, with reflected light collected by a charge-coupled device (CCD) spectrometer. Particle oxidation was controlled by Raman spectroscopy using a Witec Confocal Raman Microscope System alpha 300R (Witec, Ulm, Germany) to register the Raman spectra. Excitation in the confocal Raman microscopy was assured by a frequency-doubled Nd: YAG laser (Newport, Evry, France) at 532 nm wavelength (Figure 1).

### 2.2. Characterization of Trans-Cinnamaldehyde Loaded pSi Particles

We performed Fourier Transformed Infrared spectroscopy (FTIR) to determine if there was any chemical interaction between the pSi and TC. The TC-loaded pSi particles were taken and washed with deionized water three times and then dried the obtained particles for analysis. The spectra were collected over a range of 4000–400 cm^−1^ with 30 scans per sample. The data acquisition was performed by OPUS 7.5 (Bruker, Germany) software.

### 2.3. Trans-Cinnamaldehyde Loading and Release Kinetics

The pSi particles were loaded with trans-cinnamaldehyde (Sigma Aldrich, St. Louis, MO, USA) by passive soaking for 24 h under gentle shaking at room temperature. For loading, a 1:1 dilution of trans-cinnamaldehyde (*v*/*v*) in ethanol was used. After 24 h of incubation in loading solution, the pSi particles were rinsed in ethanol and stored in 70% ethanol solution to prevent particle degradation. Loading efficiency was assessed by UV-visible spectroscopy at a wavelength of 290 nm.

To follow the trans-cinnamaldehyde release, the particles were dried to remove the ethanol and then incubated in Phosphate Buffer Saline (PBS) (Gibco, Thermo Fisher, Waltham, MA, USA) at 37 °C. Release experiments were conducted by incubating 25 mg of TC loaded with 10 mL of PBS, i.e., 2.5 mg particles/mL of PBS. Supernatants were collected after one, four, eight, and fourteen days. The recovered samples were analyzed by High-Performance Liquid Chromatography (Waters 600E HPLC, Waters, Milford, MA, USA) with the following parameters: a 254 nm UV detector and an elution with 0.1 formic acid solution in acetonitrile at 0.5 mL/min debit. Various concentrations of TC in ethanol were used for calibration to determine the retention time (RT). For the elucidation of various kinetics models, they were tested for best fit after applying data obtained from the release study. The best-fit kinetic model was selected by comparing their R^2^_adjusted_ (R^2^_adj_) values. The model that exhibited the R^2^_adj_ value closest to one was considered the best-fitted kinetic model [25].

### 2.4. Microbial Strains and Culture Conditions

*Streptococcus mutans* UA159 and *Candida albicans* ATCC 90028 were acquired from the American Type Culture Collection (ATCC). *S. mutans* was maintained in Columbia Blood Agar (CBA) at 37 °C in anaerobic conditions (85%N_2_, 5%CO_2_, 10%H_2_), while *C. albicans* was maintained in Sabouraud Dextrose Agar (SDA) at 37 °C in aerobic conditions. For all the experiments, *S. mutans* and *C. albicans* were grown overnight in Tryptic Soy Broth (TSB) at 37 °C in a 5% CO_2_ incubator. Before each experiment, the microbial count was adjusted to 2 × 10^6^ for *S. mutans* and 2 × 10^4^ for *C. albicans* [26].

### 2.5. Microbial Growth

The effect of pSi-TC on the growth of *S. mutans* and *C. albicans* monocultures was determined by the broth microdilution assay [27]. The stock solution was prepared by dissolving pSi-TC in TSB. A working concentration of 1.15 mg/mL of pSi-TC was added to sterile 96-well polystyrene plates and serially diluted up to 0.009 mg/mL. A total of 10 µL of the inoculum was added to each well. Untreated standard cell suspensions with TSB were maintained as control. Pilot studies confirmed that pSi particles alone had no effect on microbial growth. The plates were then incubated at 37 °C in a 5% CO_2_ incubator for 24 h. The microbial growth was determined by measuring the absorbance (OD_595_) of the planktonic cells using a DTX 880 Multimode Detector (Beckman Coulter, Brea, CA, USA).

### 2.6. Biofilm Formation

The effect of pSi-TC on biofilm formation was determined directly by the well-established Crystal Violet (CV) assay. pSi-TC (100 µL) was added to TSB + 1% sucrose with 10 µL of the standard cell suspension for each of *S. mutans* and *C. albicans* and incubated at 37 °C in a 5% CO_2_ incubator for 24 h. Then, the planktonic cells were removed, and the biofilms were carefully washed twice with PBS and stained with 0.1% crystal violet. After washing with PBS, the CV stain absorbed by the biofilm biomass was retained by adding 95% ethanol, transferred to another 96-well plate, and the absorbance (OD_570_) was measured using a multimode detector.

### 2.7. Biofilm Microbial Composition

The plate count method was used to determine the proportion of *S. mutans* and *C. albicans* in the pSi-TC treated biofilms. Dual-species biofilms were developed on sterile hydroxyapatite discs (Clarkson Chromatography, PA, USA) in the presence of 0.25 mg/mL of pSi-TC based on the results of the aforementioned experiment. The treated and untreated biofilms were mechanically disrupted and dispersed in 1 mL of PBS by uniform vortexing for 1 min.

The resultant microbial suspension was then serially diluted in TSB, and a 50 µL aliquot was plated on Sabouraud Dextrose Agar (SDA) and Columbia Blood Agar (CBA) for *C. albicans* and *S. mutans*, respectively, and incubated in an anaerobic chamber and aerobic incubator, respectively, at 37 °C for 24 h. The number of viable cells was determined by counting the Colony Forming Units (CFU), which were transformed into log_10_ values.

### 2.8. Acid Production

Dual-species biofilms were developed in the presence of 0.25 mg/mL of pSi-TC. Then, the pH of the spent media was determined using a pH electrode (CyberScan pH 500, Thermo Scientific, Waltham, MA, USA), as described previously [28]. Prior to each measurement, the electrode was rinsed with deionized water and sterilized with 70% ethanol (*v*/*v*).

### 2.9. Scanning Electron Microscopic (SEM) Imaging

To directly visualize the biofilm-preventive effect of pSi-TC, biofilms were developed in the presence of pSi-TC. After 24 h of incubation, the discs were washed with PBS and fixed with 2.5% glutaraldehyde, following dehydration in ethanol series (75%, 85%, and 95% 100%). Then, the discs were gold sputter-coated and observed using SEM (Hitachi VP-SEM SU1510) to assess the biofilm architecture [29].

### 2.10. Gene Regulation Studies

The effect of pSi-TC on the biofilm and virulence-related genes was studied using Quantitative real-time Polymerase Chain Reaction analysis (qRT-PCR). Biofilms were developed in the presence of pSi-TC for 24 h. Untreated biofilms served as the negative control. After incubation, the planktonic and loosely adhered cells were removed by washing the biofilms twice with PBS. The biofilms were then removed by scraping and centrifuged at 10,000× *g* for 10 min.

RNA was extracted as per the manufacturer’s instructions and the quantity of RNA was assessed using Nanodrop. An iScript^TM^ cDNA synthesis kit was used to reverse transcribe RNA to cDNA, following which the qRT-PCR analysis was performed. The primers used for *C. albicans* and *S. mutans* are listed in Table S1. 16srRNA and ACT1 were used as the housekeeping gene for *S. mutans* and *C. albicans* respectively. The 2^-^^ΔΔCT^ method was used for calculating the gene expression changes relative to the control [11].

### 2.11. Statistical Analysis

Statistical analysis of the data was performed using Prism version 8.0.2 (GraphPad, San Diego, CA, USA). One-way ANOVA multiple comparison analysis was performed to compare the significance between the control and treatment groups, while the Mann-Whitney U-test was applied to the compare the mean pH values between the groups. *p* < 0.05 was considered statistically significant.

## 3. Results and Discussion

### 3.1. Characterization of pSi Particles

The antimicrobial and anti-biofilm efficacy of essential oils is masked in low concentrations as they are relatively unstable and easily degradable [30]. Thus, to enhance their anti-microbial efficiency by increasing the bioavailability of TC, we loaded them onto the pSi-particles. Porous silicon and its derivative particles are biocompatible and biodegradable [18]. pSi-derived microparticles and nanoparticles are considered “penetration enhancing agents”, which enhance the membrane penetration potential of hydrophobic drugs that simultaneously increases their bioavailability [31]. These properties of pSi make it an ideal carrier for the delivery of sparingly soluble essential oils [31,32,33]. In this work, we first synthesised P-type porous silicon wafers that were ultrasonicated to yield the pSi-particles. The acquired pSi particles were then evaluated for particle size, pore diameter, and porosity.

**Figure 2:**
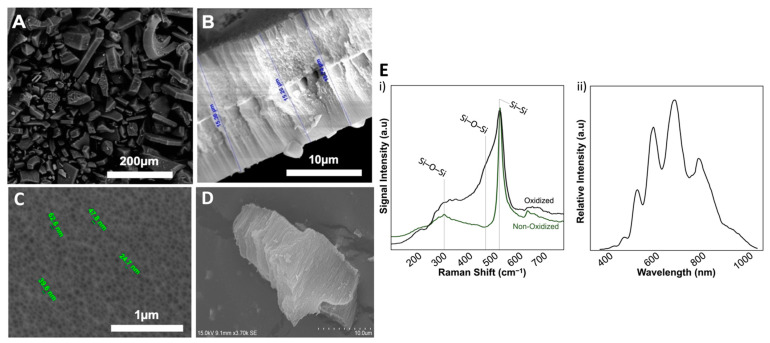
(**Left** panel) SEM images of pSi-TC particles (**A**) showing a cubic shape (×130), (**B**) side view, showing porous structure (×10,000). (**C**) top view, revealing the homogeneous porous structure; magnification ×100,000 (**D**) cross-section view of Cubic shaped pSi-TC nanoparticles. (**Right** panel, **E**): (i) Raman spectra acquired before and after oxidation of pSi samples (non-oxidized and oxidized, respectively). Oxidation is indicated by the appearance of Si-O-Si peaks (ii) IRS of pSi samples (before particle formation by sonication).

SEM examination revealed a mean pore diameter of 31 ± 11 nm with a homogeneous distribution on the surface (Figure 1). The particles were roughly cubic, with a mean edge size of 27 ± 19 µm. Interferometric reflectance spectroscopy (IRS) showed that the global porosity ranged between 70–75%, with the whole particles harbouring a porous structure. The prepared pSi particles were then thermally oxidized to prevent the surface degradation in aqueous media [30]. An increase in oxidation efficiency was confirmed by following the augmentation of broad O-Si-O peaks under Raman spectroscopy at 320 cm^−1^ (scissoring vibration) and at 468 cm^−1^ (bending vibration) (Figure 2). This oxidation procedure increased the stability and solubility of the particles [16,34].

### 3.2. Drug Loading and Release Kinetics

The assessment of TC loading by UV-Vis spectroscopy revealed an average TC loading of 45% in pSi particles (*w*/*w*). In addition, the maximal possible loading determined according to porosity (70–75%), molecular weight (MW_Cinnam_ = 132 g/mol), density (d_Cinnam_ = 1.05), and dilution of pSi-TC particles. According to this, the maximal theoretical loading was found to be 0.3 g TC/g of particles.

**Figure 3:**
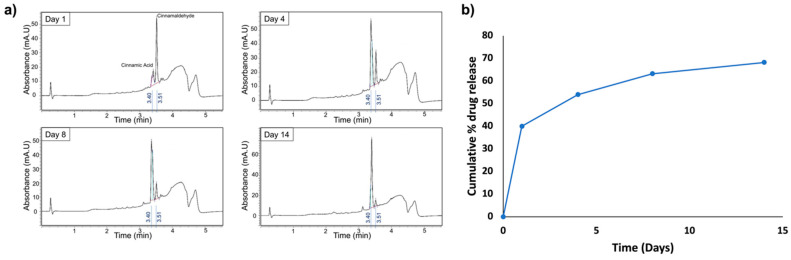
HPLC analysis of *trans*-cinnamaldehyde release over time. (**a**) Analysis of supernatants with representative chromatograms after one, four, eight, and fourteen days. The peak at 3.40 min and 3.51 min correspond to cinnamic acid and *trans*-cinnamaldehyde respectively. *Trans*-cinnamaldehyde release is maximal after one day, and decreases over time, with a slight release still visible after 14 days. (**b**) Cumulative drug release pattern of pSi-TC NPs, depicting that pSi-TC NPs able to deliver TC over a 14-day period of sustained release manner.

HPLC was used to track the drug release kinetics in PBS over 14 days of analysis at four time points (Figure 3). Samples of supernatants collected after day 1, day 4, day 8 and day 14 were analyzed by HPLC for TC concentration based on the determined Retention Time, and according to the area under the curve. HPLC calibration allowed to determine Retention Time RT = 3.51 min for TC and RT = 3.40 min for cinnamic acid, with the calibration curve according to the concentration. This concentration can be proportionally related to the area under the curve according to Beer-Lambert law, thus permitting the assessment of even small concentrations.

**Figure 4:**
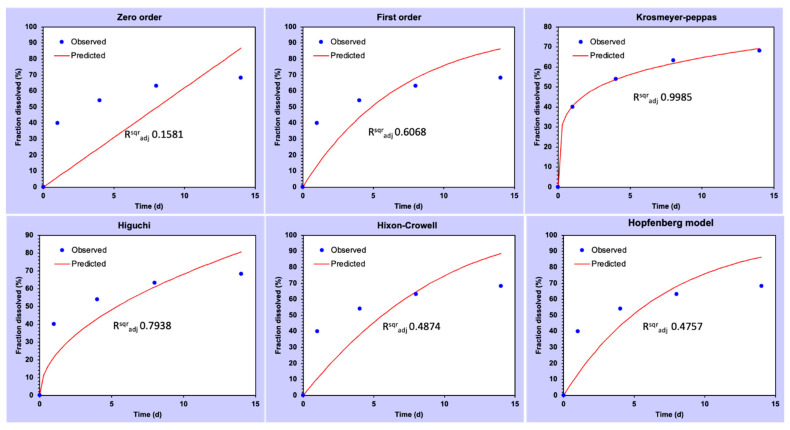
Graphs showing different drug release kinetic models for pSi-TC nanoparticles during the 14 days.

The TC concentrations recovered at the different time points were C_d1_ = 10 mg/L, C_d4_ = 3.5 mg/L, C_d8_ = 2.3 mg/L and C_d14_ = 1.2 mg/L, as measured with the 3.51 min peak. Another peak was observed at 3.40 min, corresponding to cinnamic acid, a degradation product of cinnamaldehyde (oxidized form of cinnamaldehyde). The concentration of cinnamic acid increased over time, while the cinnamaldehyde concentration decreased. Thus, the drug release kinetics showed a sustained and controlled release of TC from the pSi over a period of 14 days. Nevertheless, from the kinetics modelling (Figure 4) it has been observed that the pSi-TC exhibited Korsmeyer–Peppas kinetic as the best-fit model (R^2^_adj_) = 0.998, *n* = 0.228, which confirmed that the pSi-TC formulation followed a pseudo-Fickian transport [35].

### 3.3. FTIR-Spectra of Trans-Cinnamaldehyde Loaded pSi Particles

**Figure 5:**
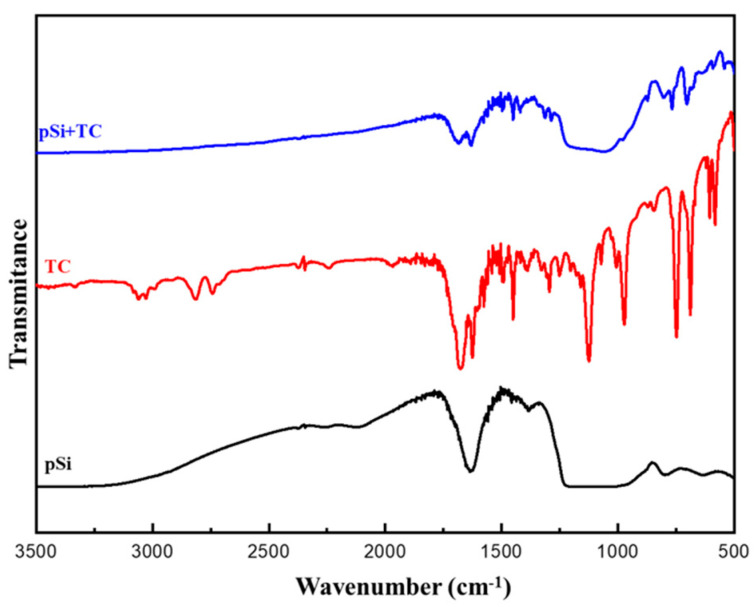
FTIR spectrum of pSi, TC and TC-loaded pSi.

The surface functional groups and chemical interaction of pSi and the pSi-TC were evaluated by FTIR spectroscopy (Figure 4). The broad band at 1083.19 cm^−1^ is attributed to Si-O-Si asymmetric stretching; the sharp peaks at 796 cm^−1^, 964.99 cm^−1^, and 470.82 cm^−1^ correspond to the O-Si bond, observed in the spectrum of pristine pSi particles [36]. In the pure TC spectrum, 3008 cm^−1^ is ascribed to the aromatic C–H bond; 2924 cm^−1^ due to the =C–H bond; 2854 cm^−1^ is assigned to the C–H bond of the carbonyl groups [25]. It was observed that in the TC-loaded pSi particles, all the peaks remained intact, and the 3008 cm^−1^ and 2924 cm^−1^ peaks were suppressed because of the high-intensity pSi particles (Figure 5). Thus, from the FTIR-spectra it is evident that pSi-particles are stable after the TC loading and there is no strong covalent chemical bond between the pSi and TC, thereby facilitating drug release.

### 3.4. Sub-Inhibitory Concentration of pSi-TC Potently Inhibits Biofilms and Acid Production

**Figure 6:**
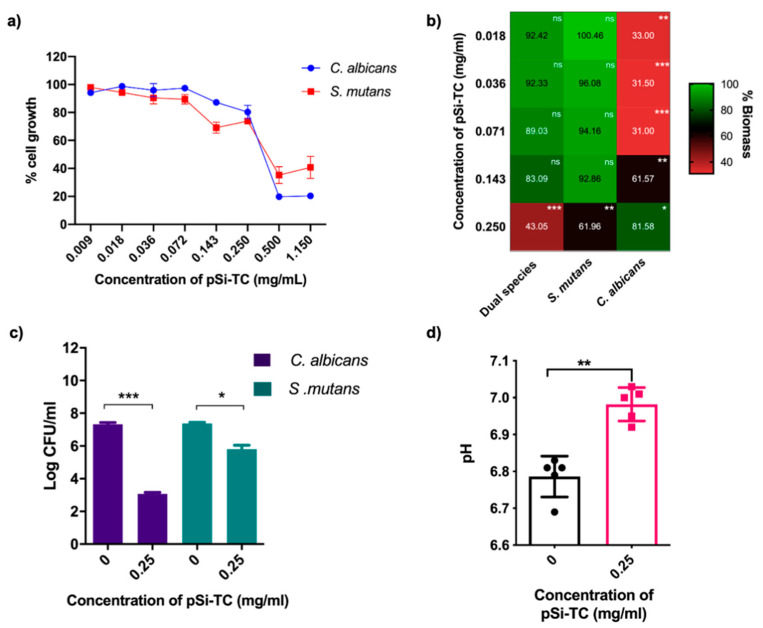
Effect of pSi-TC on (**a**) planktonic *S. mutans* and *C. albicans* cultures. Concentrations ranging from 1.15–0.009 mg/mL were tested and showed a reduction in growth at 0.5 and 1.15 mg/mL for both *S. mutans* and *C. albicans* when compared to the control, (**b**) heatmap representing the biomass of *S. mutans*, *C. albicans* mono-species and in combination, showing significant reduction in biomass when compared to control (**c**) microbial composition of the dual-species biofilm: Log_10_ changes in a number of *C. albicans* and *S. mutans* after treatment, shows a significant reduction in cell number, (**d**) pH of the spent media showing the reduced acid accumulation when compared to control, denoted by neutral pH when treated with pSi-TC. *** denotes *p* ≤ 0.0005, ** denotes *p* ≤ 0.005, * denotes *p* ≤ 0.01, ns denotes not significant *p >* 0.05.

Microbial growth studies showed that 0.5 and 1.15 mg/mL pSi-TC inhibited *S. mutans* by 78% and 85%, respectively, and 97% and 98%, respectively, for *C. albicans* (Figure 6a). Concentrations below 0.5 mg/mL exhibited minimal growth inhibition of both species. It has been shown previously that cinnamaldehyde at >0.5 mg/mL and 0.6 mg/mL is lethal to *S. mutans* [37] and *C. albicans* [38,39], respectively. The as-prepared pSi particles had no effect on planktonic cells (Figure S1). Most pathogens develop an antimicrobial resistance to conventional antimicrobials as these drugs target the growth of the microbes rather than the virulence pathways [40]. Furthermore, thwarting biofilm assembly could be a more promising approach to caries prevention than indiscriminate microbicidal activity. Therefore, we interrogated if sub-inhibitory concentrations of pSi-TC, i.e., concentrations below 0.5 mg/mL, could inhibit biofilms.

**Figure 7:**
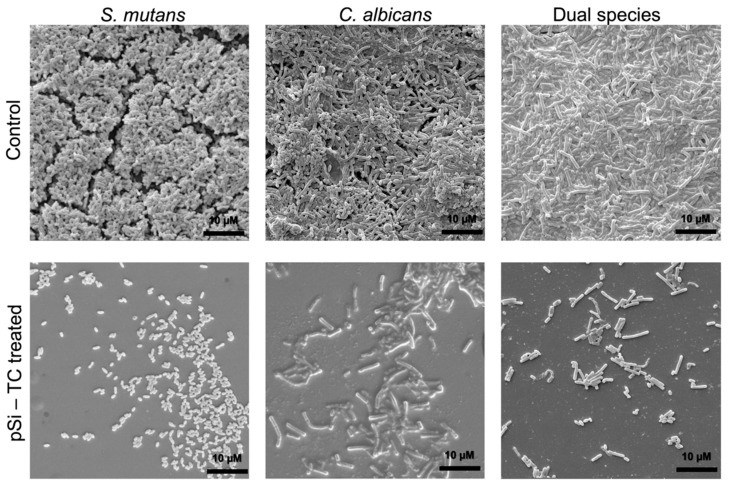
Scanning Electron Microscopic examination of biofilms of *S. mutans*, *C. albicans* and Dual-species biofilms with and without treatment with pSi-TC, showing dense biofilm aggregates with a thick biofilm matrix in the untreated control, while pSi-TC treated biofilms show scattered microbial cells and an overall reduction in the number of cells, indicating no biofilm formation.

Notably, 0.25 mg/mL significantly reduced biofilm development (*p* ≤ 0.05) in comparison to the control, whereas in *S. mutans* and *C. albicans* mono-species biofilms, a 50% biomass reduction was observed at concentrations of 0.25 mg/mL and 0.009 mg/mL, respectively (Figure 6b). Additionally, pSi-TC resulted in a 4.3 log reduction in *C. albicans* and a 1.5 log reduction in *S. mutans* (Figure 6c) within the biofilms developed on hydroxyapatite discs. Taken together, these results indicate that the reduction in biofilm biomass by pSi-TC is due to a reduction in the cell numbers, as the cells were no longer held together by the biofilm matrix. This hypothesis is supported by the microscopic images, wherein biofilms developed in the presence of biofilm inhibitory concentrations of pSi-TC for dual- (Figure 7) and mono-species biofilms (Figure 7) showed scarce and scattered *S. mutans* and *C. albicans* cells with no dense aggregates or matrix. On the other hand, the control group showed dense aggregates of cross-kingdom biofilms, held together by the extracellular matrix.

In cariogenic biofilms, a low pH due to acid production causes tooth demineralization which eventually leads to irreversible cavity formation [41]. In oral microbial communities, *S. mutans* can efficiently ferment a wide range of sugars via the glycolytic pathway, thereby releasing lactic acid. Such acid production is further enhanced in presence of *C. albicans* [41,42,43]. Our results showed a moderate, but significant increase (*p* ≤ 0.005) in the pH of the spent media, indicating decreased acid production within biofilms that were exposed to pSi-TC (Figure 6d). pSi-TC also reduced lactic acid production by 25.11% in dual-species biofilms (Figure S2).

The concentration of TC in 0.25 mg/mL of pSi-TC is 87.5 µg/mL. This is an important finding since a six-fold reduction in the TC concentration was sufficient to inhibit dual- and mono-species biofilms of *S. mutans* compared to 500 µg/mL of TC needed to inhibit *S. mutans* biofilms and acid production as reported previously [11]. The improvement in the antibiofilm and anti-virulence efficacy of TC may be attributed to its reduced hydrophobicity when encapsulated in pSi-particles, thereby increasing its availability in biofilms. Furthermore, oxygenating the pSi particles has been shown to increase the wettability of hydrophobic compounds [15,16]. Therefore, it is apparent that the controlled drug-releasing mechanism and close microbial interactions of pSi-TC are effective against dual-species cross-kingdom biofilms containing *S. mutans* and *C. albicans.* Taken together, our phenotypic results clearly demonstrate that pSi-TC prevented biofilm development and inhibited its caries-related virulence phenotype (acid production).

### 3.5. Gene Expression Analysis

**Figure 8:**
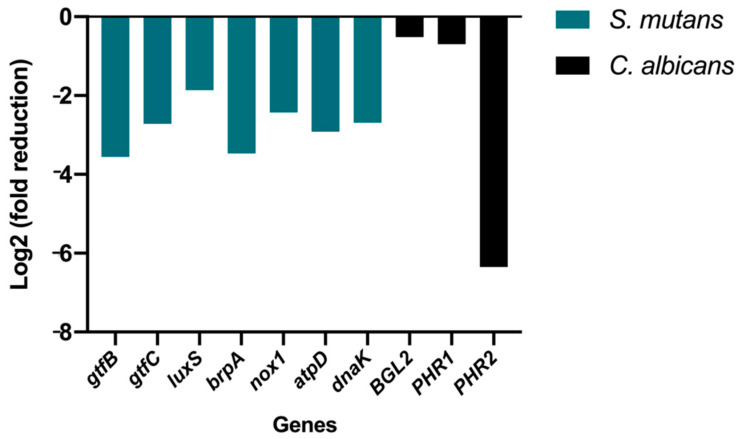
Gene expression in *S. mutans* and *C. albicans* after treatment with pSi-TC.

To understand the underlying mechanism of these biofilm-inhibitory and acid production-reducing responses of pSi-TC, gene expression analysis was performed (Figure 8). qRT-PCR analysis showed that pSi-TC significantly downregulated (*p* < 0.05) the *S. mutans* genes that regulate inter-species communication and biofilm development (~1.5 fold) (*LuxS*), genes governing heat and acid-induced stress (>2.5 fold) (*DnaK* and *AtpD*) [11], and oxidative stress tolerance (two-fold) (*Nox1*) [44]. The LuxS/AI-2 signalling is a major quorum-sensing pathway in *S. mutans* and controls interspecies biofilm formation, acid tolerance, and stress tolerance mechanisms [45], which were significantly downregulated (*p* < 0.05), corroborating strongly with our phenotypic results.

Notably, pSi-TC also significantly downregulated the genes that encode for Glucosyl transferases (>2.5 fold) (Figure 8). The genes *GtfB* and *GtfC* contribute markedly to glucan production, which forms the bulk of the EPS matrix. In addition, these genes also regulate biofilm development and facilitate bacterial adhesion to the salivary pellicle and microcolony formation, which further enhance the adhesion of *C. albicans* to the biofilms [46,47]. In fact, it has been shown that a *GtfB* mutant strain of *S. mutans* was unable to synergize with *C. albicans*, preventing the development of serious carious lesions in animal models [42]. Furthermore, *C. albicans* also produces glucans, which are regulated by *Bgl2*, *Phr1* and *Phr2* genes, all of which were significantly downregulated by pSi-TC [48,49,50]. *C. albicans* also activates *S. mutans GtfB* to produce more glucans, forming a well-organized biofilm model [45]. By reducing the number of *C. albicans* cells by >4 log in dual-species biofilms, it is likely that pSi-TC prevented the synergism between *S. mutans* and *C. albicans* by inhibiting the glucan synthesis which leads to decreased adhesion to the substrate.

While we did not characterise the cytotoxicity in this study, there is ample evidence from us and others, to support that pSi and TC are both remarkably safe compounds. The biocompatibility of pSi to several cell types has been demonstrated previously by us [51,52,53] and others [54]. In fact, pSi is considered so cytocompatible, that it is a recommended scaffold for the adhesion and proliferation of stem cells. Well-established evidence also shows that pSi is non-toxic, bioresorbable, and biodegradable. It degrades into silicic acid, which is a major form of silicon in the human body [51,55]. Similarly, TC is a food-derived constituent and is categorised by the U.S. FDA as Generally Recognised as Safe (GRAS). We [11] and others [56] have previously reported the excellent biocompatibility of TC to macrophages at concentrations significantly greater than those used in this work.

A limitation of this work was that we used a representative cross-kingdom biofilm that is typical of early childhood caries. While the use of microbes from two kingdoms strengthens the validity of the results, it does not recapitulate the complexity of the oral ecosystem and the caries microbiome, considering that oral biofilm houses more than 700 microbial species. Thus, further studies will interrogate the effects of pSi-TC on complex biofilm models in the presence of salivary conditions to better replicate the host environment. Such studies will provide insights into how pSi-TC can modulate microbial ecology.

## 4. Conclusions

This pioneering in vitro study demonstrated that:

1. Porous silicon particles are an innovative and effective approach to carrying and releasing TC.
2. pSi-TC successfully inhibited the development of cross-kingdom biofilms of *S. mutans* and *C. albicans* and its acid production.

The newly developed pSi-TC holds remarkable promise to be further developed as a caries-preventive agent.

## Data Availability

The data are available through a personal request to the corresponding authors.

## Supplementary Materials

The following supporting information can be downloaded at: https://www.mdpi.com/article/10.3390/pharmaceutics14071428/s1, Table S1: Primer list for dual-species biofilms of *S. mutans* and *C. albicans*; Figure S1: Effect of the as-prepared porous silicon microparticles on *S. mutans* and *C. albicans*; Figure S2: Effect of pSi-TC on lactic acid accumulation.
