# Supplementary material for: Trans-Cinnamaldehyde Eluting Porous Silicon Microparticles Mitigate Cariogenic Biofilms
Source: Pharmaceutics. 2022 Jul 7;14(7):1428. doi: 10.3390/pharmaceutics14071428 (PMC9322055; doi:10.3390/pharmaceutics14071428)
Supplement: Supplementary file 1 [file pharmaceutics-14-01428-s001.zip › pharmaceutics-1781839-supplementary.pdf]

Table S1: Primer list for dual species biofilms of *S. mutans* and *C. albicans*. 16srRNA and ACT1 were used as the house keeping gene for *S. mutans* and *C. albicans* respectively.

S.no	Gene	Forward Primer Sequence (5′ – 3′)	Reverse Primer Sequence (5′ – 3′)	Ref
1	*gtfB*	AAACAACCGAAGCTGATAC	CAATTTCTTTTACATTGGGAAG	[1]
2	*gtfC*	GGTTTAACGTCAAAATTAGCTGT ATTAGC	CTCAACCAACCGCCACTGTT	[2]
3	*LuxS*	ACTGTTCCCCTTTTGGCTGTC	AACTTGCTTTGATGACTGTGGC	[2]
4	*brpA*	GGAGGAGCTGCATCAGGATTC	AACTCCAGCACATCCAGCAAG	[2]
5	*nox1*	GGACAAGAATCTGGTGTTGA	CAATATCAGTCTCTACCTTAGG C	[1]
6	*atpD*	CCAGGCGGTTCATTCATCTGAC	GGCGGGATTTCGGTATTTACTG	[2]
7	*DnaK*	GGTACAACAAACTCAGCAGTTGC AGTTCTT	CCCCATCTTAGATTTGATGGAA AGAATTGT	[2]
8	*16srRNA*	CCTACGGGAGGCAGCAGTAG	CAACAGAGCTTTACGATCCGA AA	[2]
9	*BGL2*	ATGGGTGATTTGGCTTTCAA	CAGCTGGACCAAGGTTTTGT	[1]
10	*PHR1*	GGTTTGGTTCTGGTTGATGG	AGCAGCAGTTCCTGGACATT	[1]
11	*PHR2*	CTCCTCCATTTCCAGAACCA	CGTCTGAATCAACCTTGTCG	[1]
12	*ACT1*	ATTCGGTGAGTAATCCTA	GTATAGTCCAGATAACAACA	[1]

References

1. Lobo, C.I.V.; Rinaldi, T.B.; Christiano, C.M.S.; de Sales Leite, L.; Barbugli, P.A.; Klein, M.I. Dual-species biofilms of *Streptococcus mutans* and *Candida albicans* exhibit more biomass and are mutually beneficial compared with single-species biofilms. *J. Oral Microbiol.* **2019**, *11*, 1581520. https://doi.org/10.1080/20002297.2019.1581520.
2. Balasubramanian, A.R.; Vasudevan, S.; Shanmugam, K.; Lévesque, C.M.; Solomon, A.P.; Neelakantan, P. Combinatorial effects of trans-cinnamaldehyde with fluoride and chlorhexidine on *Streptococcus mutans*. *J. Appl. Microbiol.* **2021**, *130*, 382–393. https://doi.org/https://doi.org/10.1111/jam.14794.

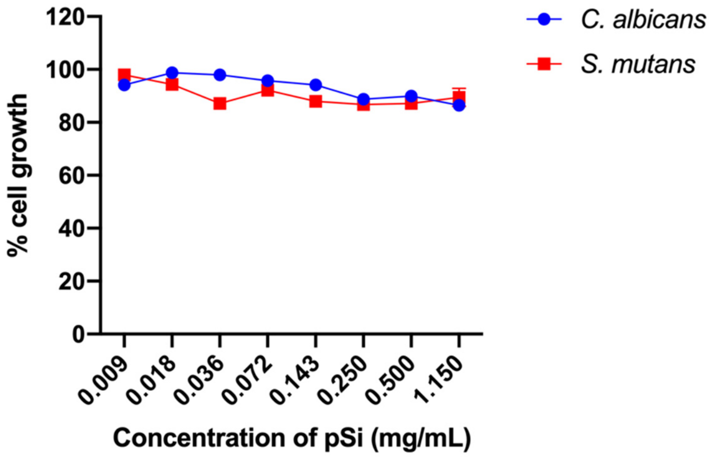

Figure S1.: Effect of the porous silicon on the planktonic growth of *S. mutans* and *C. albicans.* Concentrations ranging from 1.15-0.009 mg/ml were tested against the mono species cultures of *S. mutans* and *C. albicans,* the results showed that the pSi alone did not show any effect of the growth of the organisms when compared to control.

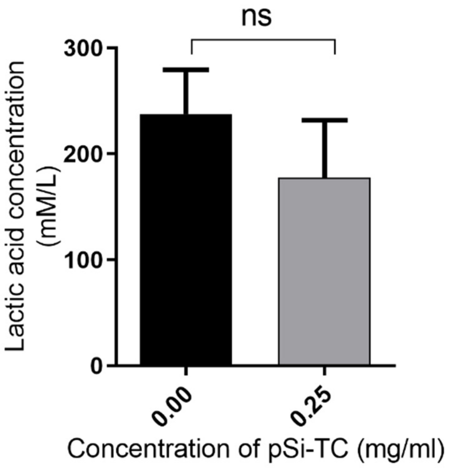

Figure S2.: Effect of pSi-TC on lactic acid accumulation. pSi-TC reduced lactic acid accumulation in dual-species biofilms by 25.11%, when compared to the control. ns denotes not significant $p > 0.05$.

Filename:	pharmaceutics-1781839-supplementary.docx
Directory:	E:\7.7\pharmaceuticals-1793104
Template:	C:\Users\MDPI\AppData\Roaming\Microsoft\Templates\Normal.dotm
Title:	
Subject:	
Author:	shanthini
Keywords:	
Comments:	
Creation Date:	7/6/2022 11:42:00 PM
Change Number:	9
Last Saved On:	7/7/2022 4:48:00 PM
Last Saved By:	MDPI
Total Editing Time:	6 Minutes
Last Printed On:	7/7/2022 4:48:00 PM

As of Last Complete Printing

Number of Pages: 2

Number of Words: 284 (approx.)

Number of Characters: 1,990 (approx.)
